# Self-reported genital warts among sexually-active university students: a cross-sectional study

**DOI:** 10.1186/s12879-018-2954-7

**Published:** 2018-01-15

**Authors:** Silvia Cocchio, Chiara Bertoncello, Tatjana Baldovin, Alessandra Buja, Silvia Majori, Vincenzo Baldo

**Affiliations:** 1 Department of Cardiac, Thoracic and Vascular Sciences, Public Health Unit, University of Padua, Padua, Italy; 2 Department of Public Health and Community Medicine, Hygiene and Environmental, Occupational and Preventive Medicine Division, University of Verona, Verona, Italy

**Keywords:** Genital warts, Human papillomavirus, Sexually-transmitted diseases

## Abstract

**Background:**

Genital warts are one of the most common forms of sexually-transmitted disease, but their epidemiology has yet to be thoroughly elucidated. The present study was designed to shed light on the prevalence of clinically-confirmed, self-reported genital warts **(**GWs) in a representative sample of the university population.

**Methods:**

In 2015, a cross-sectional survey was conducted on 11,096 individuals approached at the Students Information Bureau where they came to enroll for a university degree course. Participants completed an anonymous, self-administered questionnaire providing information on their sociodemographic characteristics, sexual behavior, and any history of clinically-diagnosed genital warts. Multivariate logistic regression was then used to identify any factors associated with the disease.

**Results:**

Our analysis was conducted on 9259 questionnaires (83.4%). Participants were a mean 21.8 ± 4.8 years of age, and 59.6% were female. Overall, 124 individuals (1.3%, 95%CI: 1.0–1.6) reported having been diagnosed with genital warts: 48 men (1.3%, 95%CI: 0.9–1.6), and 76 women (1.4% 95%CI: 1.1–1.7). Overall, 22.5% of the sample were vaccinated (1.3% of the males and 36.8% of the females). The group of respondents aged 30 years or more had the highest incidence of genital warts (males: 5.6%, 95%CI: 2.5–8.6; females: 6.9%, 95%CI: 3.4–10.4). The independent risk factors associated with a history of disease were (for both genders) a history of other sexually-transmitted diseases, and ≥2 sex partners in the previous 24 months. A protective role emerged for routine condom use. Additional risk factors associated with genital warts in males concerned men who have sex with men, bisexuality vis-à-vis heterosexuality, and smoking.

**Conclusions:**

The findings emerging from our study help to further clarify the epidemiology of genital warts in young people, and may be useful to public health decision-makers. This study showed that genital warts occur in men as well as women, and suggests that both genders should be monitored for this disease to ascertain the effects of the free HPV vaccination offered to all girls in the Veneto in their 12th year of life since 2008, and to all boys of the same age since 2015.

## Background

Genital warts (GWs) are classified as a clinical form of human papillomavirus (HPV) infection because they are visible lesions in the form of single or multiple papules developing in the area of the vulva, perineum, anus, vagina, cervix, penis, scrotum and urethra [1, 2]. The clinical symptoms of GWs include pruritus, burning sensation, vaginal discharge, and bleeding [3]. More than 90% of these lesions are associated with HPV types 6 and 11, which usually give rise to benign changes, though they are sometimes associated with malignancies [4]: from 20% to 50% of HPV-related lesions have revealed not only HPV-6 and -11, but also co-infections with other HPV types carrying a high oncogenic risk [4, 5]. Genital warts are often not considered a serious problem, and few studies have been conducted on the quality of life of patients with these lesions. On the other hand, several reports have mentioned the negative psychological effects of this disease, such as stress, embarrassment and anxiety, which are sometimes persistent [6–10].

Genital warts are one of the most common sexually-transmitted diseases (STD), occurring particularly among young people of both genders [11]. In surveys conducted on general adult populations, the self-reported lifetime history of GWs ranged from 0.36% to 12% in women [12–15], and from 0.27% to 7.9% in men [12, 16]. A history of GWs in the previous 12 months was reported by 1.0% to 1.9% of females in the 4 Nordic countries [13].

Australia, the USA and several countries in Europe have introduced HPV vaccination programs for girls with a view to the prevention of cervical cancer [6]. The targets of quadrivalent HPV vaccine are HPV types 6, 11, 16 and 18, so it has the potential to protect against GWs as well as precancerous lesions and malignancies in both genders [17–19].

In the Veneto region (north-east Italy), free quadrivalent HPV vaccination has been offered to all girls in their 12th year of life since 2008, and in 2015 the program was extended to include boys in their 12th year of life too. HPV vaccine remains freely accessible to these cohorts up until they turn 18 years old. It is also offered on a co-payment basis to women up until they are 46 years of age, and to men until they are 26 years old.

The paucity of data concerning the epidemiology of GWs in Italy prompted the present study, which was designed to estimate the prevalence of clinically-confirmed, self-reported GWs in a sexually-active university population and to pinpoint any risk factors associated with the disease.

## Methods

### Study population

A cross-sectional study on GWs was conducted at the University of Padua, Italy, between September and November 2015. The study population included individuals (with an age ≥ 18 years old) going to the Students Information Bureau to enroll for a university degree course.

### Data collection

A self-administered anonymous printed questionnaire were offered to each participant. To ensure confidentiality, participants were asked to seal their completed questionnaires in an envelope they were given at the same time.

The questionnaire covered several lifestyle factors, including sociodemographics (e.g. marital status, formal education), smoking, alcohol intake, condom use, sexual habits (e.g. age at time of first sexual intercourse, number of sex partners in the previous 2 years).

Information on GWs was obtained with the question, ‘Have you ever had genital warts diagnosed by a doctor or other health care professional?’ There were also questions on how long the episode(s) of GWs lasted and whether any had occurred in the previous 12 months. Details of any other sexually-transmitted infections, such as Chlamydia trachomatis, herpes simples, human immunodeficiency virus, syphilis or gonorrhea, were also requested.

Respondents who reported never having had sexual intercourse were excluded from the analysis.

### Statistical analysis

The recruiting target to obtain a precision in excess of 0.5% was estimated to be about 11,000 students, based on the assumption of 10% of non-responders and a prevalence of GWs of around 1.5%.

Descriptive statistics (including means and proportions for continuous variables, and percentages and absolute frequencies for categorical variables) were used to analyze the data by gender and age group (18–20 years, 21–24 years, 25–29 years, and 30+ years). The prevalence of GWs was estimated as the proportion of respondents who reported a clinically-confirmed diagnosis of GW at some time in their lives. The χ^2^ test, Student’s t-test, and 95% confidence intervals (CI95%) were used, as appropriate. Factors independently associated with the experience of GWs were examined using a multivariate logistic regression, from which adjusted ORs were estimated with corresponding 95% CIs. A *p* value < 0.05 was adopted as the threshold for statistical significance.

## Results

During the study, 11,096 of the individuals approached agreed to complete the questionnaire. After they were checked for completeness and data quality, 10,049 questionnaires (90.6%) were included; 790 subjects (7.9%) were excluded because they reported never having had sexual intercourse. The study population consisted in 9259 subjects, with a majority of females (59.6%). Participants were a mean 21.8 ± 4.8 years old (22.2 ± 5.7 for males, 21.4 ± 4.0 for females), and the vast majority (95.5%) were between 18 and 29 years old.

Table 1 shows the characteristics of the sample analyzed by gender. In 50.6% of cases, the respondents reported being in an exclusive relationship (men were less likely to do so than women, *p* < 0.001). Women were less likely than men to be single (33.6% vs 46.5%, respectively; p < 0.001). In 55.1% of the questionnaires the respondent reported using condoms. Among the female, 35.2% were using hormonal contraceptives. Our respondents reported having been a mean 17.3 ± 1.7 years old for men, and 17.3 ± 1.6 years for women (p = n.s.) at the time of their first sexual intercourse. The youngest age group considered (18- to 20-year-olds) had been younger at the time (16.8 years old) than the other age groups (18.5 years old for the respondents over thirty; *p* < 0.05). The mean number of sex partners in the previous 24 months was 1.3 ± 1.4 for the women, and 1.9 ± 2.3 for the men (*p* < 0.001). Sexually-transmitted infections other than genital warts were reported by 4.9% of the women and 3.1% of the men (p < 0.001). Overall, 22.8% of the sample reported having been vaccinated against HPV (1.3% of the males, and 36.8% of the females).

**Table 1:** Characteristics of 9259 respondents stratified by gender

Variables	Males		Females		Total	
	(N. 3742)		(N. 5517)		(N. 9259)	
Age groups [n (%)]						
18–20	1735	(46.4)	2771	(50.2)	4506	(48.7)
21–24	1477	(39.5)	2197	(39.8)	3674	(39.7)
25–29	314	(8.4)	346	(6.3)	660	(7.1)
30+	216	(5.8)	203	(3.7)	419	(4.5)
Age at time of first sexual intercourse (mean ± SD)						
18–20	16.9	(1.2)	16.7	(1.3)	16.8	(1.3)
21–24	17.3	(1.8)	17.2	(1.8)	17.2	(1.8)
25–29	17.9	(2.3)	17.8	(2.3)	17.8	(2.3)
30+	18.5	(3.5)	18.4	(2.7)	18.5	(3.1)
Sex partners in the last 24 months [n (%)]						
≤ 1	2579	(68.9)	4218	(76.5)	6797	(73.4)
≥ 2	1163	(31.1)	1299	(23.5)	2462	(26.6)
Relationship [n (%)]						
exclusive	1497	(40.0)	3187	(57.8)	4684	(50.6)
not exclusive	506	(13.5)	477	(8.6)	983	(10.6)
single	1739	(46.5)	1853	(33.6)	3592	(38.8)
Sexual orientation [n (%)]						
heterosexual	3535	(94.5)	5188	(94.0)	8723	(94.2)
homosexual	112	(3.0)	67	(1.2)	179	(1.9)
bisexual	95	(2.5)	262	(4.7)	357	(3.9)
Other sexually-transmitted infections [n (%)]						
no	3627	(96.9)	5249	(95.1)	8876	(95.9)
yes	115	(3.1)	268	(4.9)	383	(4.1)
Condom use [n (%)]						
no	594	(15.9)	1547	(28.0)	2141	(23.1)
occasionally	647	(17.3)	922	(16.7)	1569	(16.9)
yes	2318	(61.9)	2783	(50.4)	5101	(55.1)
not specified	183	(4.9)	265	(4.8)	448	(4.8)
Smoking [n (%)]						
no	2479	(66.2)	3878	(70.3)	6357	(68.7)
ex-smoker	366	(9.8)	399	(7.2)	765	(8.3)
yes	897	(24.0)	1240	(22.5)	2137	(23.1)
Alcohol intake [n (%)]						
No	491	(13.1)	1113	(20.2)	1604	(17.3)
occasionally	3030	(81.0)	4312	(78.2)	7342	(79.3)
Every day	221	(5.9)	92	(1.7)	313	(3.4)
HPV vaccination [n (%)]						
18–20	33	(0.9)	1473	(26.7)	1506	(16.3)
21–24	12	(0.3)	515	(9.3)	527	(5.7)
25–29	2	(0.1)	40	(0.7)	42	(0.5)
30+	2	(0.1)	3	(0.1)	5	(0.1)

Overall, 124 respondents (1.3%, 95%CI: 1.0–1.6) reported having been diagnosed with GWs at some time; 48 of them were men (1.3%, 95%CI: 0.9–1.6) and 76 were women (1.4%, 95%CI: 1.1–1.7). Of these 124 cases of GWs, 33 had reportedly occurred in the previous 12 months (15 cases in males, 18 in females). The episodes of GWs had lasted for a mean 3.9 ± 2.3 months, with 50% of cases cured within 3 months, 29% within 6 months, and 21% taking longer than six months.

**Fig. 1:**
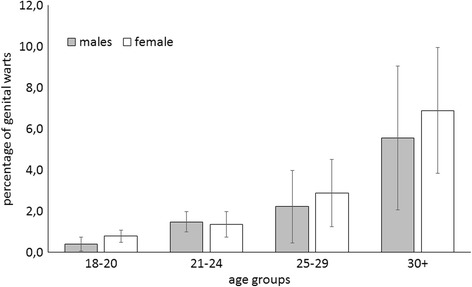
Lifetime incidence rate of self-reported genital warts by gender and age group

The mean age at the time of answering the questionnaire, and the mean number of sex partners in the previous 24 months were both significantly higher among the individuals who had experienced episodes of GWs (the GW group) than among those who had not (the no-GW group). The mean age of the GW group was 25.8 ± 8.0 years, as opposed to 21.7 ± 4.7 years for the no-GW group (*p* < 0.01). There was no statistically significant gender-related difference in the mean age of the respondents in the GW group (26.6 ± 8.2 years for men, 25.3 ± 7.9 for women). As for the mean number of sex partners, this was reportedly 3.2 ± 3.6 in the GW group, as opposed to 1.6 ± 1.8 in the no-GW group (*p* < 0.01). The mean age of the GW group at the time of their first sexual intercourse was younger than in the no-GW group (16.8 ± 1.8 vs 17.1 ± 1.7 years, respectively; *p* < 0.05). A clear pattern emerged in the likelihood of a history of GWs with increasing age (p < 0.01). In both genders, the respondents over 30 years old had the highest proportion in the GW group (5.6%, 95%CI: 2.5–8.6 for males; and 6.9%, 95%CI: 3.4–10.4 for females); the difference between men and women was not statistically significant. Fig 1 shows the rates of clinically-confirmed, self-reported GWs by gender and age.

In the GW group the mean number of sex partners in the previous 24 months was significantly higher for males than for females (3.2 ± 3.6 vs 1.6 ± 1.8, respectively; *p* < 0.05). As for sexuality, there were significantly more males than females (p < 0.05) who reported being homosexual or bisexual. Males were also significantly more likely than females (p < 0.05) to be smokers.

GWs were significantly less frequent in females who were vaccinated than in those who were not (0.6% versus 1.8%; OR 0.35 (0.19–0.64), while in males there was no significant difference between the two (Tables 2 and 3).

**Table 2:** Univariate and multivariate analysis of self-reported genital warts in males

Variables	Number of subjects	GWs		OR	Adj OR
		N	(%)	(95% CI)	(95% CI)
Age [continuous variable]	3742	48	(1.3)	1.10 (1.07–1.13)	1.05 (1.01–1.09)
Age at time of first sexual intercourse [continuous variable]				0.88 (0.76–1.02)	0.96 (0.81–1.13)
Sex partners in the last 24 months					
≤ 1	2579	14	(0.5)	reference	
≥ 2	1163	34	(2.9)	5.52 (2.95–10.3)	3.58 (1.63–7.88)
Relationship					
exclusive	1497	22	(1.5)	reference	
not exclusive	506	11	(2.2)	1.49 (0.72–3.09)	0.97 (0.40–2.36)
single	1739	15	(0.9)	0.58 (0.27–1.28)	0.69 (0.30–1.55)
Sexual orientation					
heterosexual	3535	31	(0.9)	reference	
homosexual	112	10	(8.9)	11.0 (5.30-23.1)	8.88 (3.70-21.3)
bisexual	95	7	(7.4)	8.99 (3.3-24.5)	5.16 (1.88-14.1)
Positive history of sexually-transmitted infections					
no	3627	32	(0.9)	reference	
yes	115	16	(13.9)	18.1 (9.69–34.0)	8.20 (3.95–17.0)
Vaccination status					
no	3693	47	(1.3)	reference	
yes	49	1	(2.0)	1.62 (0.22–11.9)	1.87 (0.21–16.4)
Condom use					
no	594	17	(2.9)	reference	
occasionally	647	13	(2.0)	0.70 (0.34–1.45)	0.69 (0.30–1.60)
yes	2318	18	(0.8)	0.27 (0.13–0.55)	0.40 (0.18–0.88)
not specified	183	0	(0.0)		–
Smoking (reference: no)					
no	2479	12	(0.5)	reference	
ex-smoker	366	13	(3.6)	7.57 (3.43–16.7)	6.11 (2.47–15.1)
yes	897	23	(2.6)	5.41 (2.71–10.8)	4.60 (2.05–10.3)
Alcohol intake					
no	491	12	(2.4)	reference	
occasionally	3030	30	(1.0)	0.40 (0.20–0.78)	0.30 (0.13–0.69)
yes	221	6	(2.7)	1.11 (0.46–2.70)	0.35 (0.11–1.11)

**Table 3:** Univariate and multivariate analysis of self-reported genital warts in females

Variables	Number of subjects	GWs		OR	Adj OR
		N	(%)	(95% CI)	(95% CI)
Age [continuous variable]	5517	76	(1.4)	1.06 (1.03–1.09)	1.10 (1.06–1.13)
Age at time of first sexual intercourse [continuous variable]				0.94 (0.78–1.12)	0.89 (0.77–1.02)
Sex partners in the last 24 months					
≤ 1	4218	41	(1.0)	reference	
≥ 2	1299	35	(2.7)	2.82 (1.79–4.45)	1.92 (1.09–3.36)
Relationship					
exclusive	3187	41	(1.3)	reference	
not exclusive	477	11	(2.3)	1.81 (0.92–3.55)	1.76 (0.81–3.82)
single	1853	24	(1.3)	1.01 (0.49–2.07)	1.30 (0.72–2.35)
Sexual orientation					
heterosexual	5188	70	(1.3)	reference	
homosexual	67	1	(1.5)	1.11 (0.15-8.09)	0.81 (0.11-6.19)
bisexual	262	5	(1.9)	1.42 (0.16-12.4)	0.70 (0.24-2.02)
Positive history of sexually-transmitted infections					
no	5249	55	(1.0)	reference	
yes	268	21	(7.8)	8.03 (4.79–13.5)	5.72 (3.23–10.1)
Vaccination status					
no	3486	63	(1.8)	reference	
yes	2031	13	(0.6)	0.35 (0.19–0.64)	0.50 (0.26–0.96)
Condom use					
no	1547	32	(2.1)	reference	
occasionally	922	21	(2.3)	1.10 (0.63–1.92)	0.93 (0.49–1.77)
yes	2783	23	(0.8)	0.39 (0.22–0.72)	0.47 (0.25–0.91)
not specified	265	0	(0.0)		–
Smoking (reference: no)					
no	3878	45	(1.2)	reference	
ex-smoker	399	8	(2.0)	1.74 (0.82–3.72)	0.92 (0.39-2.20)
yes	1240	23	(1.9)	1.61 (0.71–3.63)	0.91 (0.51-1.62)
Alcohol intake					
no	1113	17	(1.5)	reference	
occasionally	4312	55	(1.3)	0.83 (0.48–1.44)	1.04 (0.54–1.99)
yes	92	4	(4.3)	2.93 (1.04–8.26)	1.35 (0.37–4.85)
Oral contraceptive use					
No	3577	28	(0.8)	reference	
yes	1940	28	(1.4)	1.86 (1.1–3.14)	1.10 (0.63–1.92)

The multivariate analysis showed that, for both genders, the independent variables associated with a history of GWs were: age (adjusted OR 1.05 [1.01–1.09] in males, 1.10 [1.06–1.13] in females); ≥2 sex partners in the previous 24 months (adjusted OR 3.58 [1.63–7.88] in males, 1.92 [1.09–3.36] in females); a history of sexual infections other than GWs (adjusted OR 8.20 [3.95–17.2] in males, 5.72 [3.23–10.1] in females); while routine condom use had a protective role (adjusted OR 0.40 [0.18–0.88] in males, and 0.47 [0.25–0.91] in females). For males, additional factors independently associated with GWs were a sexuality other than heterosexuality, and smoking (Table 2), while in females there was a protective role of vaccination (adjusted OR 0.50 [0.26–0.96] (Table 3).

## Discussion

In this survey of university students (a selected population), comprising more than 10,000 young men and women (who were a mean 21.8 years old), the lifetime prevalence of clinically-confirmed, self-reported, GWs was similar between the genders, with an overall 1.3% in males and 1.4% in females. In a US population aged 18–59 years, the prevalence of a positive history of GWs was 7.2% for women, and 4% for men [15]; in a sample population aged 16–55 years in the Czech Republic, the figures were 6.1% and 5.8% in women and men, respectively [20]; in a population aged 18–45 years in Estonia, they were 4.6% and 2.8%, respectively [21, 22]; and in Denmark the figures were higher, at 10.6% in women and 7.9% in men [13]. The results of other studies on women of similar age placed their lifetime prevalence of GWs at 12% in Iceland, 11.3% in Sweden, and 9.5% in Norway [13]. The prevalence identified in our population was low by comparison with the above-mentioned studies, probably because our sample was still very young (95.5% of respondents were between 18 and 29 years old). In fact, our analysis identified a rising trend in the positive history of GWs with age in both genders, which peaked at 5.6% and 6.9% in males and females, respectively, among our respondents over 30 years old (the difference between genders was not statistically significant), reaching figures more similar to those of other studies [13, 15, 20–22]. Other reasons contributing to our lower prevalence may relate to methodological differences (e.g. study design), and to geographical and cultural differences in sexual behavior. In our study population, there was also evidence of the respondents who were younger at the time of answering our questionnaire having been younger when they first had sexual intercourse.

For both genders, the two factors most strongly associated with the risk of developing GWs were a history of other sexual infections and ≥2 sex partners in the previous 24 months, as seen in other studies [13, 15, 20–23].

Consistently with other reports [22], men who described themselves as homosexual carried a very significantly higher risk of developing GWs than men reporting to be heterosexual.

In our sample, routine condom use conferred a significant reduction in the risk of developing GWs in both genders. This finding is consistent with a retrospective case-control study on men and women attending the Sydney Sexual Health Centre, with and without newly-diagnosed GWs [24]. As for the use of hormonal contraceptives, our analysis revealed no additional risk of GWs among women using oral contraceptives. This result contradicts the findings of a study conducted on women with and without GWs seen at a city-center clinic for genitourinary medicine in Scotland, which suggested that women taking oral contraceptives may be at higher risk of presenting with GWs [25].

The influence of smoking on the risk of acquiring GWs is controversial. It is hard to say whether smoking is a confounder or a genuine risk factor for GWs: some authors found no correlation [26, 27], while our data would seem to support other reports of a positive link between smoking and the burden of GWs among males [20, 22, 13, 24, 28].

Our data should be interpreted bearing in mind some limitations of this study. We used a self-administered questionnaire in a selected population in order to maximize the participation rate and ensure a large sample size, but this means that the risk of selection and interview bias cannot be ruled out (i.e. a bias due to respondents under-reporting any behavioral risk factors, for instance).

In Italy, free anti-HPV vaccination has been actively offered to all girls in their 12th year of life since 2008, and there has been a significant reduction in the cases of GWs over time in the age groups for which the vaccination’s coverage had reached a good level [29]. Because no information was available on the timing of any self-reported HPV vaccination in our sample, it is impossible to say whether, or how the prevalence of GWs identified in this study might have been influenced by vaccination. Nonetheless, a protective role of vaccination did emerge for females, probably due to the inclusion of women who had been vaccinated at 12 years old. In the remainder of our sample not actively offered vaccination, it is likely that those who reported having had a vaccination had done so because of a known risk of contracting GWs, or after an episode of GWs.

We nonetheless judge our results to be valid and reliable for the purpose of drawing comparisons with other populations (self-reporting on GWs has been used in several other studies in Europe and the USA [15, 20–22, 13]).

To achieve a higher specificity, our question on any history of GWs referred only to clinically confirmed diagnoses. As a consequence, our figures may underestimate the real prevalence of this condition because some respondents may have had GWs but not gone to a doctor to confirm their diagnosis. Individuals who develop GWs may have a limited capacity to recognize them, or be unwilling or unable to seek treatment. There are numerous psychological and social issues involved, which may contribute to explaining why prevalence estimates tend to be higher in studies based on clinical genital examinations [1].

## Conclusion

Our study contributes to the body of knowledge on the epidemiology of HPV-related disease among young people. These findings may be useful to public health decision-makers because they provide a basis for assessing the potential burden of genital HPV that can be prevented by vaccination programs. The present study showed that not only women suffer from GWs, and suggests that both genders should be monitored for the occurrence of these lesions – also to shed light on the effects of HPV vaccination programs offered in a given region, for instance.

## Abbreviations

CI95%: Confidence intervals
GW: Genital warts
HPV: Human papillomavirus
STD: Sexually-transmitted diseases
